# Skeletal muscle phosphatidylcholine and phosphatidylethanolamine respond to exercise and influence insulin sensitivity in men

**DOI:** 10.1038/s41598-018-24976-x

**Published:** 2018-04-25

**Authors:** Sindre Lee, Frode Norheim, Hanne L. Gulseth, Torgrim M. Langleite, Andreas Aker, Thomas E. Gundersen, Torgeir Holen, Kåre I. Birkeland, Christian A. Drevon

**Affiliations:** 1 Department of Nutrition, Institute of Basic Medical Sciences, Faculty of Medicine, University of Oslo, Oslo, Norway; 2 Department of Endocrinology, Morbid Obesity and Preventive Medicine, Oslo University Hospital, Oslo, Norway; 3 Department of Medicine, Division of Cardiology, University of California at Los Angeles, Los Angeles, CA 90095 USA; 4 Vitas Ltd, Oslo Innovation Park, Oslo, Norway; 5 Institute of Clinical Medicine, Faculty of medicine, University of Oslo, Oslo, Norway

## Abstract

Phosphatidylcholine (PC) and phosphatidylethanolamine (PE) composition in skeletal muscle have been linked to insulin sensitivity. We evaluated the relationships between skeletal muscle PC:PE, physical exercise and insulin sensitivity. We performed lipidomics and measured PC and PE in *m. vastus lateralis* biopsies obtained from 13 normoglycemic normal weight men and 13 dysglycemic overweight men at rest, immediately after 45 min of cycling at 70% maximum oxygen uptake, and 2 h post-exercise, before as well as after 12 weeks of combined endurance- and strength-exercise intervention. Insulin sensitivity was monitored by euglycemic-hyperinsulinemic clamp. RNA-sequencing was performed on biopsies, and mitochondria and lipid droplets were quantified on electron microscopic images. Exercise intervention for 12 w enhanced insulin sensitivity by 33%, skeletal muscle levels of PC by 21%, PE by 42%, and reduced PC:PE by 16%. One bicycle session reduced PC:PE by 5%. PC:PE correlated negatively with insulin sensitivity (β = −1.6, *P* < 0.001), percent area of mitochondria (ρ = −0.52, *P* = 0.035), and lipid droplet area (ρ = 0.55, *P* = 0.017) on EM pictures, and negatively with oxidative phosphorylation and mTOR based on RNA-sequencing. In conclusion, PC and PE contents of skeletal muscle respond to exercise, and PC:PE is inversely related to insulin sensitivity.

## Introduction

Type 2 diabetes mellitus (T2DM) affects ~415 million adults worldwide, and is predicted to rise to ~642 million by 2040 (http://www.diabetesatlas.org). Approximately 12% of global health expenditures are spent on diabetes (http://www.diabetesatlas.org). T2DM is characterized by hyperglycemia and strongly associated with insulin resistance, adiposity, and physical inactivity^1–3^.

Physical activity is known to improve insulin sensitivity and glucose tolerance^4,5^, which might prevent development of T2DM. A single bout of exercise is sufficient to increase skeletal muscle glucose uptake for several hours^6^. Twelve weeks of exercise can significantly improve whole-body glucose metabolism and insulin sensitivity^7^. Extensive improvement in insulin sensitivity may occur by combining strength- and endurance training^8,9^. Many pathophysiological variables influence insulin sensitivity such as muscle mass, maximum oxygen uptake (VO_2_max), many signal molecules, and tissue fat content^7^.

Skeletal muscle phosphoacylglycerol composition and different classes of phospholipids are related to membrane fluidity, lipid rafts^10^, membrane-protein dynamics and insulin receptor kinetics^11–13^. Phosphoacylglycerols are also important for structural integrity of mitochondria, membrane potential, substrate transport, Ca^2+^ homeostasis, morphological changes in organelles^14^, and insulin sensitivity^15–17^.

Phosphatidylcholine (PC) and phosphatidylethanolamine (PE) are the major phospholipids in cellular membranes. PC accounts for ∼50%, whereas PE represents 20–30% of the total phospholipid pool^18^. Studies on skeletal muscle-specific knock-out models of PC- and PE-related enzymes^19–21^ have shown reduced PE synthesis and increased PC:PE ratio, in addition to reduced skeletal muscle mass, endoplasmic reticulum/sarcoplasmic reticulum (ER/SR) Ca^2+^ ATPase (SERCA) activity, and low exercise performance^19–21^. Thus, there might be a role for the PC:PE ratio in skeletal muscle growth, contraction, exercise performance, and glucose metabolism. Studies on humans with obesity or T2DM, as well as endurance-trained athletes, suggest a role for the PC:PE ratio in skeletal muscle metabolism and insulin sensitivity^22^.

The aim of our study was to evaluate the skeletal muscle PC:PE ratio in response to long-term physical exercise, as well as potential links between changes in the PC:PE ratio and improved insulin sensitivity. We conducted an intervention with an acute bicycle test before as well as after 12 w of combined strength- and endurance training^7^ to assess changes in insulin sensitivity and the skeletal muscle PC:PE ratio in normal weight control men and dysglycemic overweight men.

## Methods

**Figure 1:**
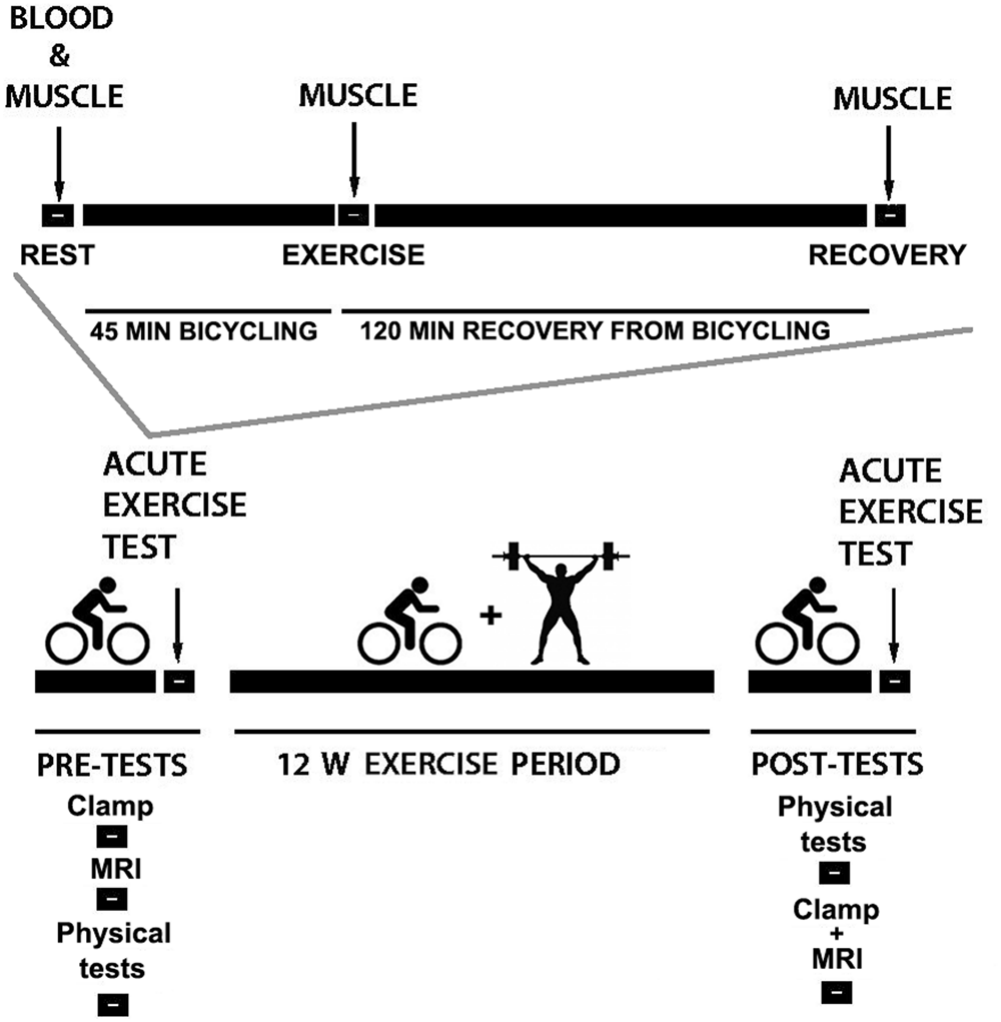
Study design of MyoGlu. Participants underwent several tests before and after the 12 w combined strength- and endurance-exercise intervention. The pre-tests (clamp → MRI → strength/VO_2_max → acute bicycle challenges) and post-test (strength/VO_2_max → clamp + MRI → acute bicycle challenges) were standardized on preceding activity and rest. Blood and muscle samples were obtained during the acute bicycle challenges at 70% of VO_2_max (top panel).

Extensive details regarding the MyoGlu study^7,23^ are published elsewhere, and core information is provided below. The study design of MyoGlu is presented in Fig. 1.

### Study participants

Twenty-six sedentary men were recruited in (1) a normoglycemic, normal weight group (n = 13), and (2) a dysglycemic overweight group (n = 13). In some previous articles from this study, we excluded two men in each group because they differed slightly from the predefined criteria for BMI and glycemia; group 1: F-glucose <5.6 mmol/L and 2 h glucose <7.8 mmol/L and BMI 19–25; group 2: F-glucose ≥5.6 mmol/L and/or 2 h glucose ≥7.8 mmol/L and BMI 27–32^7^. Hence, two of 13 subjects in group 1 had BMI slightly above 25, but were insulin sensitive documented by the clamp. Two of 13 subjects in group 2 had glucose levels just below the cut-offs, but were insulin resistant documented by the clamp. In the present study we compared the results excluding (11 vs. 11) or including (13 vs. 13) these subjects, with no differences in conclusions. Thus, we present the results including all subjects (n = 26).

### Diet and exercise

Participants registered their habitual diet in an extensively validated food frequency questionnaire before and after intervention^24–27^. Food composition and energy intake were similar during the intervention based on calculated intake using the food database AE-10 and KBS food and nutrients calculation system (KBS Version 7.1, 2013). Alcohol intake was not allowed to exceed two units per day. During testing at baseline and after 12 w the participants consumed a standardized meal after an overnight fast. All men were sedentary before inclusion to the study (<1 exercise session/w for the previous year). A carbohydrate-rich meal including ~2 slices of bread (“Ingers Superrug” from “Bakers,” the amount was adjusted depending on individual energy requirement), apple juice, cheese (total of 30 g fat), and jam, providing 23% of estimated total daily energy expenditure and ingested 90–120 min prior to the bicycle tests and sampling of biopsies. The bicycle tests were typically performed in the morning; the standardized meal was the only intake after overnight fast. Water could be consumed freely.

### Exercise intervention

#### Acute bicycle tests

An acute bicycle test was performed before as well as after the 12 w exercise intervention. After 10 min warm-up, the participants cycled 45 min at an individual workload equivalent to 70% of their personal VO_2_max. For the post-test a new workload was calculated corresponding to the post-test VO_2_max. Participants refrained from strenuous physical activity two days before the test, and had the last standard endurance session three days before post-tests.

#### Strength and endurance training

The participants underwent combined resistance and endurance exercise for 12 w, including two endurance bicycle sessions (60 min each) and two whole-body resistance-training sessions (60 min each) per week. All exercise sessions were supervised by trained personnel.

Resistance-training included leg press, leg curl, chest press, cable pull-down, shoulder press, seated rowing, abdominal crunches, and back extension for three sets each. A linear progression was followed; 4 w with 12 repetitions maximum (RM), 4 w with 10 RM and 4 w with 8 RM, with progressively increased loads. Abdominal crunches and back extension were performed with 12–20 repetitions the whole period.

Endurance-training included one session of 7 min intervals at 85% of maximum heart rate (HR_max_), and one session of 2 min intervals at >90% of HR_max_ per week. Rests between intervals included either slow cycling or complete rest. The number of 7 min intervals progressed from 3 to 4 after the second week, and from 4 to 5 after 6 weeks. The number of 2 min intervals progressed from 6 to 7 after the second week, and from 7 to 10 after 6 weeks.

### Physical fitness and insulin sensitivity

#### VO_2_max

The VO_2_max tests have been described in detail elsewhere^7^. Briefly, the tests started at a workload similar to the final load of an incremental test in which the relationship between work and oxygen uptake was established. After 1 min cycling, the workload was increased by 15 watts every 30 s until exhaustion. Success was defined as reaching an oxygen uptake plateau (<0.5 mL·kg^−1^·min^−1^ increase after 30 watt increased workload), respiratory exchange ratio (RER) values above 1.10, and blood lactate >7.0 mmol/L. The subjects abstained from training two days before the VO_2_max tests.

#### Euglycemic hyperinsulinemic clamp

Euglycemic hyperinsulinemic clamp^7,28^ was performed after overnight fast. Insulin sensitivity is reported as glucose infusion rate (GIR) relative to body weight (mg/kg/min) during the last 30 min of the clamp. A fixed dose of insulin 40 mU/m^2^·min^−1^ was infused, and glucose 200 mg/mL was adjusted to maintain plasma glucose levels at 5.0 mmol/L for 150 min. Whole blood glucose concentration was measured by a glucose oxidase method (YSI 2300, Yellow Springs, OH), and plasma glucose was calculated as whole blood glucose × 1.119. No physical exercise was performed within 3 days prior to the test.

### Skeletal muscle biopsy sampling

Skeletal muscle biopsies (n = 154) were taken from *m. vastus lateralis* using a modified Bergström procedure^29^; (1) at rest, (2) immediately after 45 min sub-max test, and (3) 2 h after ended sub-max test, before as well as after the 12 w intervention. For some participants the 1^st^ and 2^nd^ biopsies were obtained from the same incision site, but from opposite angles. After sterilization, the subcutaneous and superfacial tissues were injected with Xylocain-adrenaline 10 mg/mL + 5 μg/mL, and when deemed necessary by the physician, the actual muscle tissue was injected with a non-adrenalin anesthetic (Lidokain, 10 mg/mL). A 6 mm (diameter) muscle biopsy needle (Pelomi, Albertslund, Denmark) was used with a 50 mL syringe for vacuum generation. Muscle biopsies were quickly rinsed in cold PBS and dissected on a cold aluminum plate to remove blood and subcutaneous adipose tissue before freezing.

### Skeletal muscle lipidomics

Quantification of lipid classes in accurately weighed muscle biopsies, freeze-dried and stored at −80 °C. At the day of analysis the samples were thawed and homogenized in chloroform, methanol and water (65:35:0.4) using a motorized pellet pestle (Kontes; Vineland, NJ, USA). After centrifugation the supernatants were analyzed for lipid class content using an Agilent 1100 normal phase liquid chromatographic system coupled to an Evaporative Light Scattering Detector (ELSD). Separation of the neutral lipids was performed on a Chromolith Performance Si 100–4.6 mm HPLC column (Merck, Darmstadt, Germany) using a mixture of hexane, tert-butyl methyl ether and acetic acid (1000:50:0.25; vol) as mobile phase. The polar lipids were separated on an YMC PVA-SIL-NP 250 × 4.6 mm, 5 µm column using hexane, isopropanol, acetonitrile, chloroform, water, tert-butyl methyl ether and acetic acid mobile phase A (431:457:42:42:20:9:1.5; vol), and mobile phase B (333:507:42:42:70:7:0.15; vol) with gradient elution. Unknowns were calibrated against known standards form NU-CHEK-PREP, INC, MN, USA and reported as g/100 g sample. Standards used for PE were lipoid GMBH, PN: 585009-2120002-01/900, 1,2-diacyl-sn-glycero-3-phosphoethanolamine, and for PC were lipoid GMBH, PN: 579209-2120002-01/974, phosphatidylcholine from soybean.

### Electron microscopy

Selected bundles of muscle biopsies were taken just after acute exercise before as well as after the training intervention (n = 18), and handled as previously described^23^. Briefly, the tissue was embedded in Durcupan, ultrathin sections of 60 nm were cut using an ultramicrotome from Leica (Vienna, Austria), and images were obtained using a Tecnai G2 electron microscope from FEI (Hillsboro, OR, USA). Point counting^30^ was performed at 6000‐fold magnification on 5 randomly selected areas, total of 400 μm^2^, from each subject using a 150 × 150 lattice. The hit points of lipid droplets/mitochondria were marked manually on blinded images, and counted. The percentage was estimated by the ratio of hit points/total points*100%.

### RNA isolation, cDNA synthesis, TaqMan real-time RT-PCR and mRNA sequencing

Frozen biopsies were cooled in liquid nitrogen and crushed to powder by a pestle in a liquid nitrogen-cooled mortar. Frozen biopsies were transferred into 1 mL QIAzol Lysis Reagent (Qiagen, Hilden, Germany), and homogenized using TissueRuptor (Qiagen) at full speed for 15 sec, twice. Total RNA was isolated from the homogenate using miRNeasy Mini Kit (Qiagen). RNA integrity and concentration were determined using Agilent RNA 6000 Nano Chips on a Bioanalyzer 2100 (Agilent Technologies Inc, Santa Clara, CA). Using High‐Capacity cDNA Reverse Transcription Kit (Applied Biosystems, Foster, CA), 200 ng of total RNA was converted to cDNA for TaqMan real‐time RT‐PCR. The cDNA reaction mixture was diluted in water and cDNA equivalent of 25 ng RNA used for each sample. Quantitative real‐time PCR was performed with reagents and instruments from Applied Biosystems in the 96‐well format using a 7900 HT Fast instrument and the SDS 2.3 software (Applied Biosystems). A predeveloped primer and probe set (TaqMan assays; Applied Biosystems) was used to analyze mRNA levels of Peroxisome Proliferator‐Activated Receptor Gamma, Coactivator 1 Alpha (PPARGC1A, Hs01016719_m1). Relative target mRNA expression levels were calculated as 2−ΔCt, and normalized to beta‐2 microglobulin (B2M, Hs00984230_m1). mRNA sequencing was performed using the Illumina HiSeq 2000 system (Illumina, San Diego, CA). cDNA sequenced reads alignment was carried out using Tophat v2.0.8. Reads counted by gene feature were performed by featureCounts in Rsubread 1.14.2 and analyzed using DEseq2^31^.

### MRI

Briefly, the ankle-to-neck MRI protocol included a 3D DIXON acquisition providing water and lipid quantification^7^. Water and lipid images were derived from multi-echo data using the vendor’s inline post processing, and further processed using the nordicICE software package (NordicNeuroLab, Bergen, Norway). We measured total body volume, and thigh muscle area (from 15 cm above the knee joint space).

### Gene Set Enrichment Analysis (GSEA)

Pathway analyses were used to study the association between the PC:PE ratio and a large number of mRNAs, by correlating the PC:PE ratio with the expression of genes belonging to pre-defined, well-studied signaling pathways. We performed overlap tests based on hypergeometric means to explore pathways associated with the PC:PE ratio. The number of genes significantly correlated with the PC:PE ratio was compared to the total number of genes in a pathway using a hypergeometric distribution, which tests whether more genes in a pathway correlate with the PC:PE ratio than expected by chance. The *P*-values were calculated from a hypergeometric distribution in R using the syntax phyper (k-1, K, N-K, n), where K = genes in pathway; N = total number of genes tested; n = total number of significantly correlated genes; and k = significantly correlated genes in a pathway. The hypergeometric *P-*value is calculated as the probability of randomly drawing k or more successes from the population in n total draws. Sets of transcripts tested for enrichment were taken from the MSigDB database (KEGG gene sets; http://software.broadinstitute.org/gsea/msigdb/). We considered results as significant if the false discovery rate (FDR) was <0.05^32^.

### Statistics

Data were analysed using linear (mixed) regressions^33^ and presented as means ± SEM. We tested hypotheses concerning group differences at baseline, interaction effects between groups and time, the overall effect of exercise and group-specific exercise responses. Correlation analyses were performed using Pearson’s r or Spearman’s rank, as appropriate. Lipidomics data were lacking for one subject and the data was imputed using NIPALS^33^, with no influence on effect estimates or conclusions based on significance level. We evaluated normality and homogeneity by visual inspection of plots of residuals against fitted values. *P*-values were considered significant at α = 0.05, but should be interpreted with care due to the explorative nature of this study and the large number of tests performed. All data were analyzed using R (R Development Core Team, 2009).

### Ethics approval

The MyoGlu study adhered to the Declaration of Helsinki and was approved by the National Regional Committee for Medical and Health Research Ethics North, Tromsø, Oslo, Norway. Written informed consent was obtained from all participants prior to any study-related procedure.

## Results

### Subject characteristics and responses to exercise

**Figure 2:**
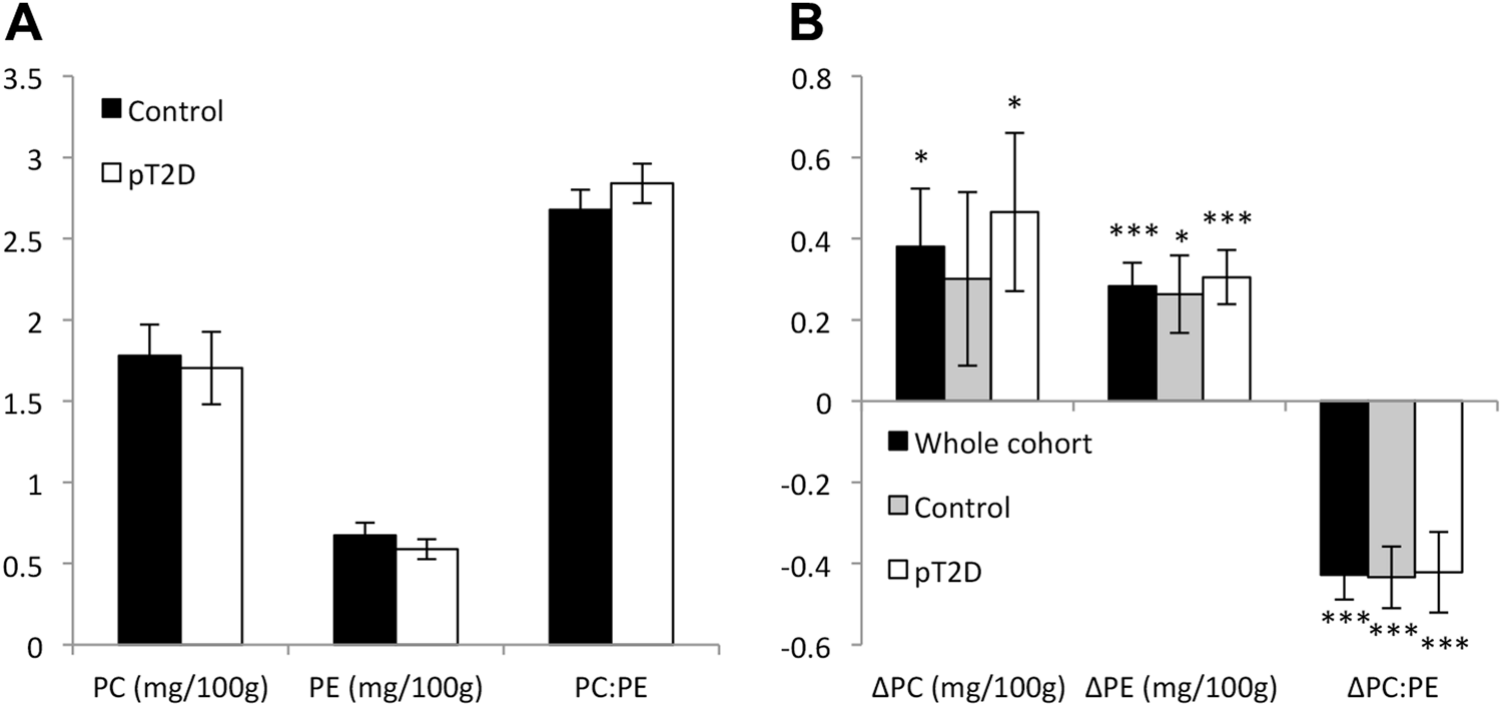
Skeletal muscle PC and PE levels and the PC:PE ratio. (**A**) Resting levels of PC and PE, and the PC:PE ratio in skeletal muscle from dysglycemic overweight men (pT2D; n = 13) and normal weight control men (Control; n = 13). (**B**) Changes in PC- and PE-levels, and the PC:PE ratio in response to 12 w exercise among all participants combined (n = 26, black bars), and in the two groups separately (n = 13, grey and white bars). Units are indicated on the x-axis, not the y-axis, because the PC:PE ratio is by definition without an unit. Data represent means ± SEM. *P < 0.05 and ***P < 0.0001 for the change being zero. Data were analyzed using linear (mixed) regression and presented as mg/100 g wet weight.

In the MyoGlu study the dysglycemic men had lower GIR and VO_2_max than the control men, but higher body weight, body volume, BMI, plasma glucose and insulin levels, leg press strength and larger thigh muscles as compared to control men at baseline (Table 1). Skeletal muscle levels of PC and PE and the PC:PE ratio were comparable between dysglycemic and control men (Fig. 2A).

**Table 1:** Subject characteristics in the MyoGlu study at baseline and after 12 w exercise intervention^1^.

	Baseline			Δ (after – before)		
	All	Control	pT2D	All	Control	pT2D
Body weight (kg)	86.9(12.5)	78.5(8.2)	95.4(10.2)^*^	−1.0(2.0)^$^	−0.3(1.6)	−1.7(2.2)^$^
BMI (kg/m^2^)	26.3(3.5)	23.5(2.0)	29.0(2.4)^*^	−0.2(0.9)	0.0(0.5)	−0.4(1.2)
Body volume (L)	75.6(11.6)	67.3(7.0)	83.9(8.9)^*^	−0.8(2.5)	0.0(1.8)	−1.5(2.9)^$^
Thigh muscle area (AU)	22.2(3.5)	20.3(2.9)	24.0(3.1)^*^	1.8(1.1)^$^	1.9(0.7)^$^	1.6(1.4)^$^
VO_2_max (mL/kg/min)	40.6(5.8)	44.1(4.4)	37.1(4.9)^*^	5.2(3.4)^$^	5.7(4.1)^$^	4.8(2.8)^$^
Leg press (kg)	224.1(41.4)	199.6(36.9)	248.7(30.3)^*^	24.0(15.9)^$^	18.5(12.5)^$^	29.6(17.3)^$#^
Leg press/tma	10.0(1.2)	9.6(1.1)	10.4(1.2)	0.3(0.9)	0.0(0.7)	0.5(1.0)^$#^
GIR (mg/kg/min)	5.9(2.4)	7.6(1.6)	4.2(1.8)^*^	2.0(1.8)^$^	2.7(2.0)^$^	1.2(1.1)^$^
F-glucose (mmol/L)	5.6(0.5)	5.4(0.5)	5.9(0.3)^*^	0.1(0.3)^$^	0.2(0.2)^$^	0.1(0.4)
F-insulin (pmol/L)	51.9(26.6)	38.5(18.6)	65.3(27.1)^*^	6.0(24.7)	0.2(20.3)	11.7(28.1)^#^

^1^GIR: glucose infusion rate; FFM: fat free mass; AU: arbitrary units; ^*^P < 0.05 compared to control, ^$^*P* < 0.05 compared to baseline, ^#^*P* < 0.05 interaction effect, corrected for baseline differences. *N* = 13 men in each group. Data represent means (SD). pT2D; dysglycemic overweight men. tma = thigh muscle area.

Combined strength and endurance exercise for 12 w enhanced GIR, VO_2_max, leg press strength, and muscle size in both groups (Table 1). Furthermore, 12 w exercise caused appreciable alterations in skeletal muscle PC- and PE-levels, and in the PC:PE ratio (Fig. 2B). Both PC- and PE-levels were increased (21 and 42%, respectively), whereas the PC:PE ratio was reduced 16% in the whole cohort (Fig. 2B, black bars). Similar alterations were observed in both groups separately (Fig. 2B, grey and white bars).

### Effect of acute exercise before and after 12 w of exercise intervention

**Figure 3:**
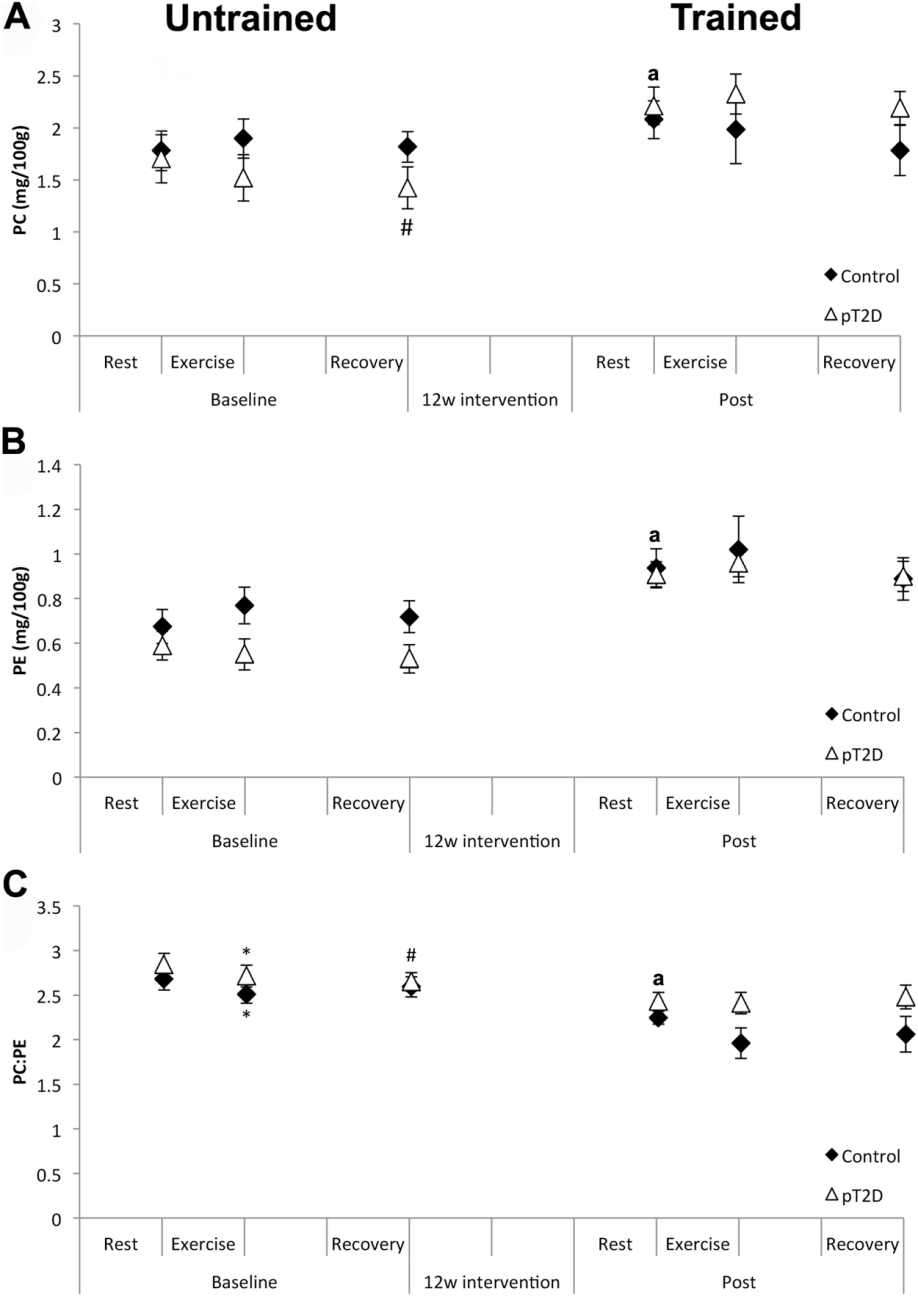
PC- and PE-levels, and the PC:PE ratio in skeletal muscle after acute exercise in the untrained and trained state. (**A**) PC- and (**B**) PE-levels and the (**C**) PC:PE ratio were measured at rest, after 45 min cycling and after 2 h recovery, before (untrained, left) as well as after (trained, right) 12 w exercise intervention. Comparisons were performed using linear (mixed) regression. pT2D = dysglycemic overweight men; control = normal weight men. Data represent means ± SEM. *P < 0.05; rest vs. exercise, ^#^*P* < 0.05 rest vs. recovery. ^a^Resting values of PC and PE were increased, whereas the PC:PE ratio was decreased between post and baseline, as presented in Fig. 2B.

Skeletal muscle PC- and PE-levels, and the PC:PE ratios were measured at rest, just after 45 min cycling at 70% of VO_2_max and after 2 h recovery following the acute exercise at baseline as well as after 12 w of exercise intervention (Fig. 3A–C). Whereas no clear patterns were observed for PC and PE levels individually, the PC:PE ratio was reduced in both groups after acute exercise (Fig. 3C). This reduction was observed at baseline in the untrained state, but not after 12 w exercise intervention in the trained state (Fig. 3C).

### Skeletal muscle PC:PE ratio may predict insulin sensitivity

**Figure 4:**
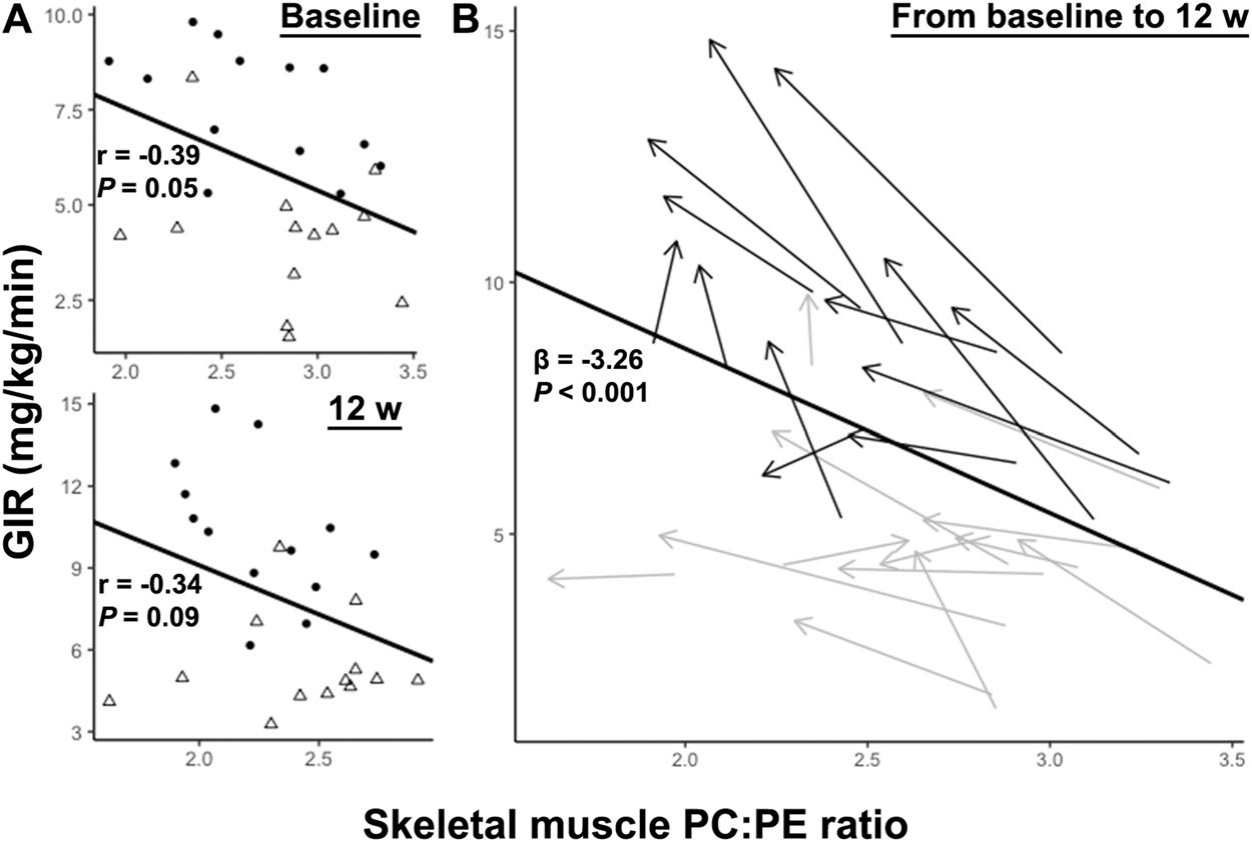
Prediction of insulin sensitivity based on the skeletal muscle PC:PE ratio. (**A**) Negative correlations between the skeletal muscle PC:PE ratio and GIR were observed at baseline and after 12 w exercise intervention across all men. Between-subjects Pearson’s correlations at baseline (top) and after 12 w of intervention (bottom) are presented. pT2D = open triangles; control = black dots. (**B**) Individual changes in skeletal muscle PC:PE ratios and GIR during the training period^45^. pT2D = grey arrows and control = black arrows indicating the change from baseline to 12 w. Arrow lengths indicate the magnitude of change.

The PC:PE ratio trended to correlate negatively with insulin sensitivity between-subjects (Fig. 4A) and reached significance within-subjects (Fig. 4B).

### Potential mechanisms of altered PC- and PE-levels

**Figure 5:**
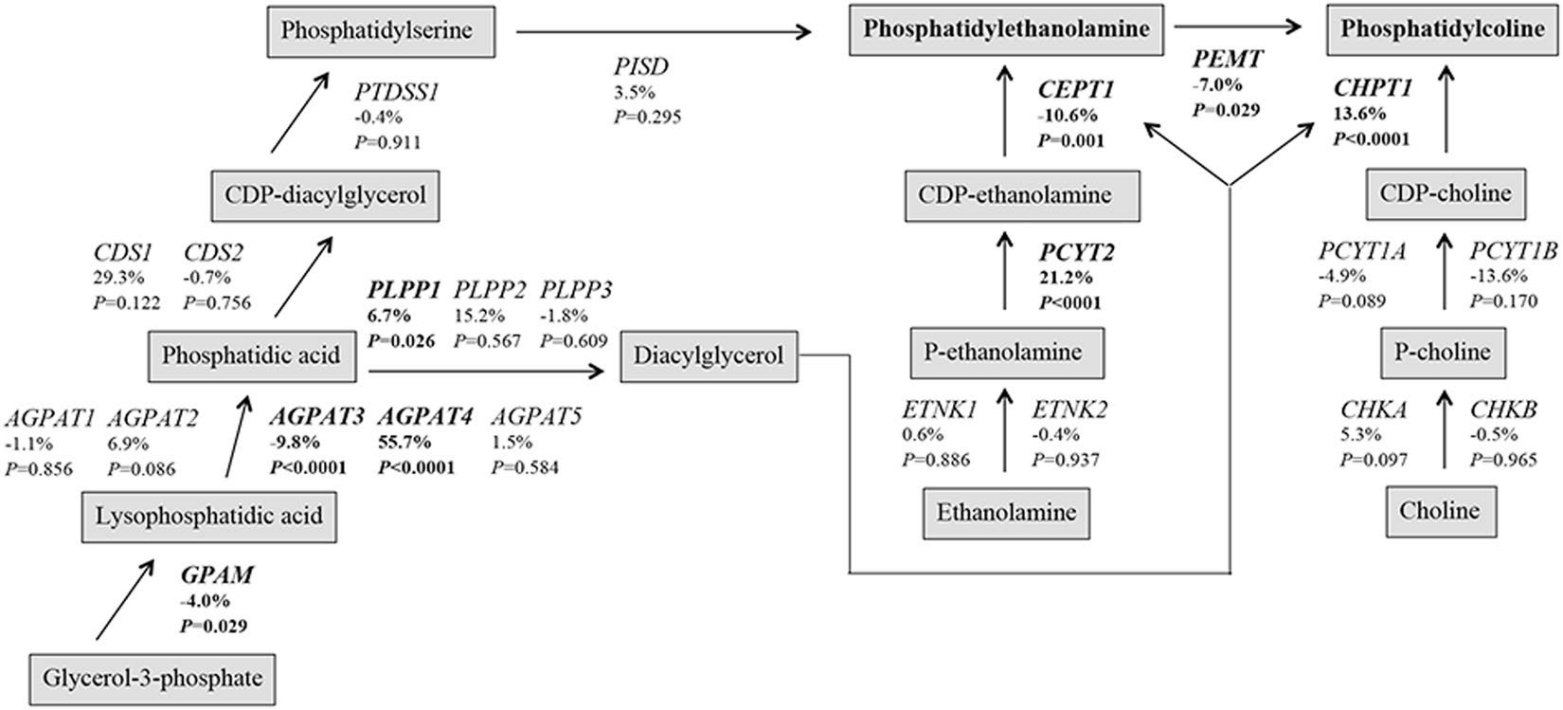
Comparison of skeletal muscle transcripts based on RNA-Seq for enzymes in the PC and PE biosynthetic pathways in response to 12 w exercise intervention. Changes in the glycerolphosphate and Kennedy pathways were analyzed using DESeq2^31^. *P*-values are reported along with %-changes in response to 12 w exercise. Significant changes (*P* < 0.05 and FDR < 0.1) are bolded. AGPAT, 1-acylglycerol-3-phophate acyltransferase; CDS, CDP-diacylglycerol synthase; CEPT, choline/ethanolamine phosphotransferase; CHK, choline kinase; CHPT1, choline phosphotransferase; ETNK, ethanolamine kinase; GPAM, glycerol-3-phosphate acyltransferase; PCYT1, choline phosphate cytidylyltransferase; PCYT2, ethanolamine phosphate cytidylyltransferase; PEMT, phosphatidylethanolamine *N*-methyltransferase; PISD, phosphatidylserine decarboxylase; PLPP, phospholipid phosphatase; PTDSS, phosphatidylserine synthase. The results were similar analyzing the two MyoGlu groups together (shown) and separately (not shown).

**Figure 6:**
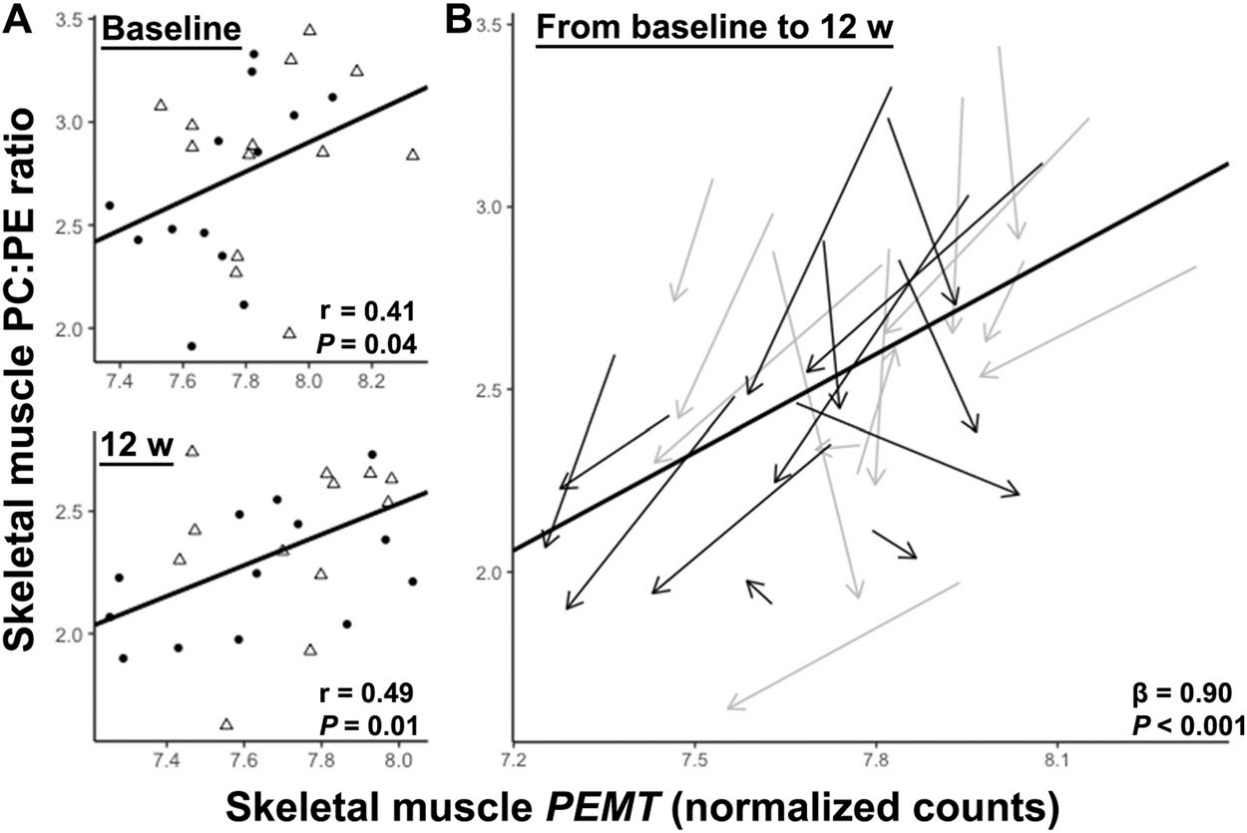
Prediction of skeletal muscle PC:PE ratios based on skeletal muscle *PEMT* mRNA transcription. (**A**) Skeletal muscle *PEMT* mRNA levels correlated positively with the PC:PE ratio both at baseline and after 12 w exercise intervention across all men. Between-subjects correlations at baseline and after 12 w of exercise intervention are presented. pT2D = open triangles; control = black dots. (**B**) Individual changes in skeletal muscle *PEMT* mRNA levels and PC:PE ratios during the training period^45^. pT2D = grey arrows and control = black arrows indicating the change from baseline to 12 w. Arrow lengths indicate the magnitude of change.

We observed no concerted increase in phospholipid synthesizing pathways after 12 w exercise intervention, based on mRNA expression (Fig. 5). However, some enzyme mRNAs were responsive to exercise; eg. expression of the enzyme *PCYT2* in the PE synthetic pathway and the enzyme *CHPT1* in the PC synthetic pathway were increased, whereas the expression of the *PEMT* enzyme, responsible for synthesis of PC from PE, was reduced. We did not observe any consistent changes in gene expression within the glycerol phosphate pathway. Furthermore, the PC:PE ratio correlated with *PEMT* expression (Fig. 6) and *PISD* expression (baseline: ρ = −0.53, *P* = 0.007, delta: ρ = −0.53, *P* = 0.001). We did not observe any consistent changes in PC- and PE-related transcripts after acute exercise (data not shown).

### Potential links between the PC:PE ratio and insulin sensitivity

**Figure 7:**
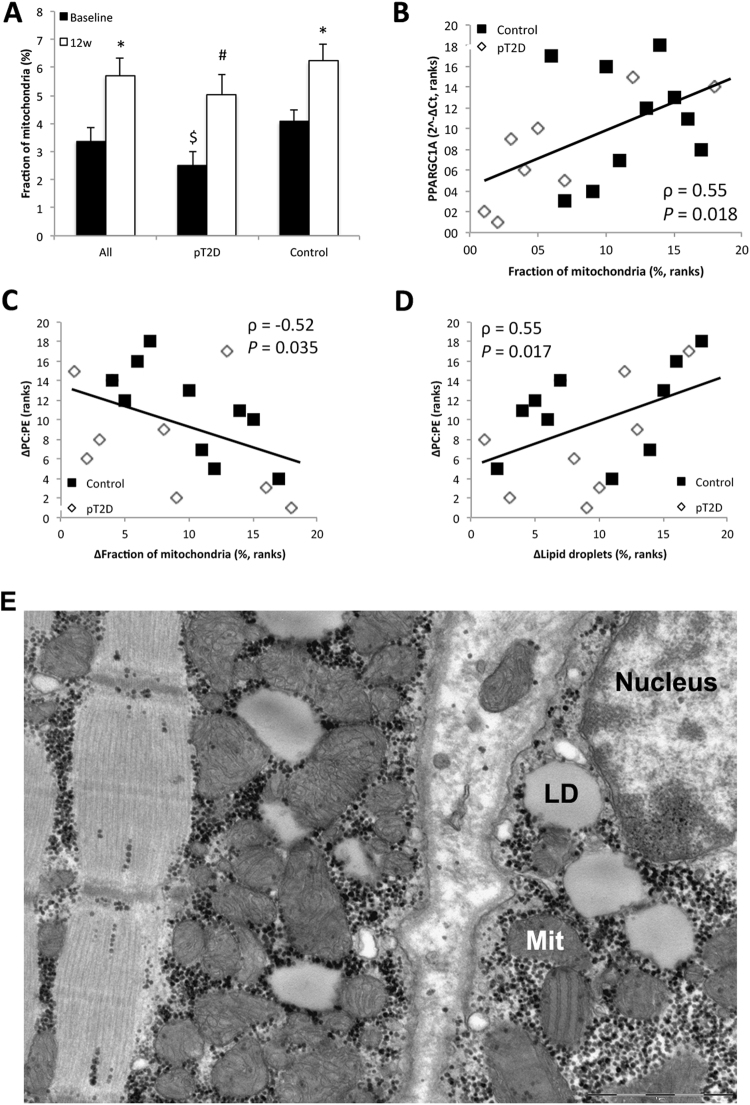
Percent (%) area of skeletal muscle cells covered by mitochondria. (**A**) The % area of mitochondria before and after 12 w exercise intervention. (**B**) The % area of mitochondria correlated with mRNA levels of PGC-1α at baseline. (**C**) Changes in the % area of mitochondria in response to 12 w exercise correlated negatively with changes in the PC:PE ratio. (**D**) Changes in the % area of lipid droplets in response to 12 w exercise correlated with changes in the PC:PE ratio. (**E**) Mitochondria (Mit) and lipid droplets (LD) in skeletal muscle cells were quantified from electron micrographs using point counting. See methods for details. Two skeletal muscle fibers are depicted interspersed with extracellular matrix. Mitochondria exhibit cristae, and LDs and grains of glycogen are seen. Eight dysglycemic overweight men (pT2D) and 10 normal weight (control) men were available for mitochondrial and LD quantification. Data represent means ± SEM. *P < 0.05 compared to baseline. ^#^*P* < 0.1 compared to baseline. ^$^*P* < 0.1 compared to control. Linear (mixed) regression and Spearman’s rank correlations were performed.

Changes in the PC:PE ratio in response to 12 w exercise intervention correlated negatively with metabolic gene expression (i.e. oxidative phosphorylation) and mTOR, based on transcriptomic analyses (Table 2). In addition, changes in the PC:PE ratio in response to 12 w exercise intervention correlated with the percent area of mitochondria and lipid droplets as compared to total cell area based on EM pictures (Fig. 7C and D). mRNA levels of PGC1α *(PPARGC1A)*, measured by RT-qPCR, correlated with the percent area of mitochondria (Fig. 7B), with a similar result using RNA-Seq to quantify PGC1α (ρ = 0.60, *P* = 0.008). The area of mitochondria increased from 3.4 to 5.4% after 12 w exercise intervention for all 18 men together (Fig. 7A). A similar increase was observed in both groups (Fig. 7A), although only the control group reached statistical significance (Fig. 7A). Additional information regarding the lipid droplets in skeletal muscles have been published elsewhere^23^.

**Table 2:** Transcriptional pathways related to the PC:PE ratio in skeletal muscle^1^.

PC:PE	K	k	k/K	*P*	FDR
Ribosome	88	14	0.1591	1.88E-7	3.49E-5
Oxidative phosphorylation	135	15	0.1111	7.43E-6	3.45E-4
Protein export	24	6	0.2500	4.5E-5	1.39E-3
MAPK signaling pathway	267	20	0.0749	9.01E-5	2.39E-3
Steroid hormone biosynthesis	55	8	0.1455	1.54E-4	3.58E-3
Primary bile acid biosynthesis	16	4	0.2500	8.96E-4	1.76E-2
Cell cycle	128	11	0.0859	1.1E-3	1.76E-2
Spliceosome	128	11	0.0859	1.1E-3	1.76E-2
Axon guidance	129	11	0.0853	1.17E-3	1.76E-2
Linoleic acid metabolism	29	5	0.1724	1.23E-3	1.76E-2
Valine, leucine and isoleucine degradation	44	6	0.1364	1.45E-3	1.8E-2
Vasopressin-regulated water reabsorption	44	6	0.1364	1.45E-3	1.8E-2
Purine metabolism	159	12	0.0755	2.04E-3	2.37E-2
Cysteine and methionine metabolism	34	5	0.1471	2.57E-3	2.81E-2
Hypertrophic cardiomyopathy (HCM)	85	8	0.0941	2.89E-3	2.85E-2
Pathways in cancer	328	19	0.0579	2.92E-3	2.85E-2
DNA replication	36	5	0.1389	3.32E-3	3.01E-2
mTOR signaling pathway	52	6	0.1154	3.46E-3	3.01E-2
Long-term depression	70	7	0.1000	3.71E-3	3.01E-2
Mismatch repair	23	4	0.1739	3.72E-3	3.01E-2
Cell adhesion molecules (CAMs)	134	10	0.0746	5.04E-3	3.78E-2
GPI-anchor biosynthesis	25	4	0.1600	5.09E-3	3.78E-2
Vascular smooth muscle contraction	115	9	0.0783	5.61E-3	4.01E-2
Glycerophospholipid metabolism	77	7	0.0909	6.28E-3	4.31E-2
Pathogenic Escherichia coli infection	59	6	0.1017	6.49E-3	4.31E-2
Pyrimidine metabolism	98	8	0.0816	6.86E-3	4.4E-2
Lysosome	121	9	0.0744	7.76E-3	4.81E-2
GnRH signaling pathway	101	8	0.0792	8.18E-3	4.9E-2
Chemokine signaling pathway	190	12	0.0632	8.43E-3	4.9E-2

^1^mRNA transcript levels negatively correlated with the change in the PC:PE ratio in response to 12 w exercise intervention overlapping with known pathways. Kegg gene sets were obtained from http://software.broadinstitute.org/gsea/msigdb/. *P*-values were calculated from a hypergeometric distribution and corrected for multiple testing using the Benjamini-Hochberg procedure (FDR; false discovery rate). GPI; Glycosyl-phosphatidyl-inositol.

## Discussion

Substantial evidence indicates that phospholipid composition is biologically important for several functions within the skeletal muscle linked to mitochondria, cell growth, contraction, exercise performance, and insulin sensitivity^14^. The main findings of our present investigation were: *1*) the skeletal muscle PC:PE ratio may predict insulin sensitivity both at baseline and in response to long-term physical exercise; *2)* the skeletal muscle PC:PE ratio is reduced in response to acute as well as long-term physical exercise; *3)* there are several potential molecular links between the skeletal muscle PC:PE ratio and insulin sensitivity; mitochondrial function might be an important one.

Alterations in skeletal muscle PC and PE concentrations are related to synthesis and degradation. Synthesis of PC and PE involves several phospholipid synthesizing enzymes involved in the glycerolphosphate and Kennedy pathways (Fig. 5). We did not observe concerted changes on the mRNA level within these two pathways in response to the exercise intervention. However, we observed increased *PCYT2* and *CHPT1* mRNA levels (Fig. 5) in parallel to the increased PC and PE levels (Fig. 2). We also observed decreased *PEMT* mRNA levels in response to 12 w exercise intervention (Fig. 5) and striking correlations with the PC:PE ratio (Fig. 6). This might explain a reduction in the PC:PE ratio after exercise intervention, because PEMT is responsible for synthesis of PC from PE. This is in line with the observation that *Pemt*^(−/−)^ mice have a reduced PC:PE ratio, increased mitochondrial activity, and high cellular ATP levels in skeletal muscle^34^.

Reduced skeletal muscle PC:PE ratio in response to 12 w exercise intervention was paralleled with increased GIR in our study (Fig. 4). GIR is a measurement of full-body insulin sensitivity, although approximately 80% variation in GIR might be explained by skeletal muscle insulin sensitivity^28^. Mitochondrial density and function in skeletal muscle may relate to exercise-induced increases in insulin sensitivity^35^. We observed that changes in skeletal muscle PC:PE ratio in response to the exercise intervention was negatively correlated with changes in skeletal muscle oxidative phosphorylation and mTOR signaling, based on transcriptomics (Table 2). Both these pathways are responsive to long-term exercise, and are associated with increased mitochondrial biogenesis, oxidative metabolism and insulin sensitivity^36^. Furthermore, changes in skeletal muscle PC:PE ratio in response to 12 w exercise intervention correlated negatively with changes in the percent area of mitochondria in muscle cells (Fig. 7). Moreover, mitochondria exhibit high abundance in skeletal muscle (Fig. 7), and mitochondrial membranes have a low PC:PE ratio (~1.2), at least compared to the sarcolemma (~2.1) and the ER/SR (~2.0) in rat *m. vastus lateralis*^37^. The inner mitochondrial membrane is most enriched with PE, and increased folding of the inner membrane might increase oxidative capacity^14^. Although we do not have direct measurements of membrane folding or enzyme activity, our data might imply that the skeletal muscle PC:PE ratio and GIR relate to the density, morphology, and function of mitochondria.

The PC:PE ratio in ER/SR membranes is increased in insulin-resistant compared to insulin-sensitive primary muscle cells^38^. Murine studies on skeletal muscle-specific knock-outs of PC- and PE-related enzymes have shown reduced PE synthesis, and increased PC:PE ratio in ER/SR membranes^19–21^. These alterations caused reduction in skeletal muscle mass, ER/SR Ca^2+^ ATPase (SERCA) activity, and exercise performance^19–21^. Because long-term exercise increases insulin sensitivity, skeletal muscle mass and exercise performance, these adaptations may be associated with alterations in ER/SR membranes. Other possible links between changes in skeletal muscle PC:PE ratio and changes in insulin sensitivity may be due to the role of PC and PE in plasma membrane integrity^39^, fluidity and lipid rafts^10^ with effects on insulin receptor kinetics^11–13^, cytokine-induced inflammation^40–42^, and glucose uptake^19–21^.

Whereas PC and PE did not respond to 45 min acute exercise at 70% VO_2_max in neither group, the PC:PE ratio was reduced after exercise in both groups at baseline but not after 12 w intervention (Fig. 3). These results apparently contrast those of Newsome *et al*.^22^. Although PC and PE exhibited marked group-dependent responses among athletes and subjects with T2DM or obesity, no changes were observed in the PC:PE ratio^22^. There are several possible reasons for these apparent differences: (a) whereas the exercise challenge was performed after overnight fast in Newsome *et al*.^22^, our participants received a standardized carbohydrate-rich meal prior to testing; (b) substrate utilization differs when testing at 50% of VO_2_max for 1.5 h^22^ compared to 70% of VO_2_max for 45 min, with increased carbohydrate oxidation at higher VO_2_max^43,44^; (c) the total duration between resting and recovery biopsies in Newsome *et al*.^22^ was 3 h and 30 min, as compared to 2 h and 45 min in our study, perhaps capturing different stages in time responses; (d) the between-group differences in Newsome *et al*.^22^ were substantially larger concerning training status and insulin sensitivity compared to our study. As of why the PC:PE ratio in our data only responded to acute exercise at baseline, but not after 12 w might be explained by long-term adaptations to exercise; such as differences in substrate oxidation^43,44,22^. Unfortunately, we do not have data from indirect calorimetry, and are thus unable to relate short-term responses in PC and PE to fat and carbohydrate oxidation.

Limitations in this study include extensive amounts of data based on mRNA expression. Thus, we can only speculate concerning flux of lipids between membranes and organelles. GIR was normalized to body weight in this study, which might be biased by changes in body composition. However, normalizing GIR to FFM, derived from body impedance measurements, did not affect the conclusions (not included).

**Figure 8:**
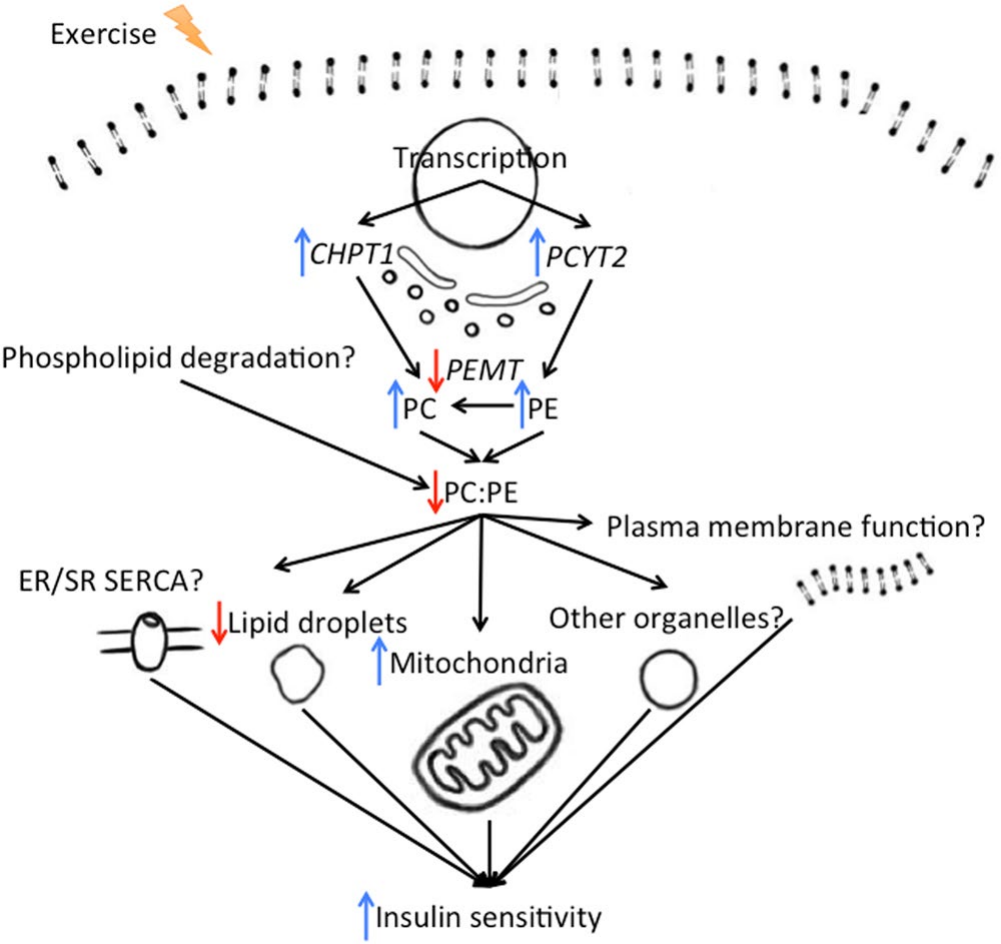
Summary of findings and potential links between physical exercise, PC, PE, and insulin sensitivity. Skeletal muscle PC:PE ratio is responsive to physical exercise, inversely related to GIR (largely reflecting skeletal muscle insulin sensitivity), and proportional to skeletal muscle transcriptional levels of phospholipid synthesizing enzyme mRNA. The skeletal muscle PC:PE ratio also correlates with intramyocellular lipid droplets and mitochondria, sarco/endoplasmic reticulum Ca^2+^-ATPase^19–21^, mRNA of oxidative enzymes in mitochondria, and plasma membrane insulin receptors^11–13^, suggesting a complex role for PC and PE in skeletal muscle insulin sensitivity.

In conclusion, the skeletal muscle PC:PE ratio are elevated in conditions with insulin resistance, and reduced with increased insulin sensitivity. The molecular link between the skeletal muscle PC:PE ratio and insulin sensitivity may involve several mechanisms related to cellular membrane alterations. A summary of the findings are presented in Fig. 8.

### Data availability

The datasets analysed during the current study are being made freely available in conjunction with a separate publication, but are available from the corresponding author on request.
