# Author Correction: Skeletal muscle phosphatidylcholine and phosphatidylethanolamine respond to exercise and influence insulin sensitivity in men

**DOI:** 10.1038/s41598-018-26061-9

**Published:** 2018-05-15

**Authors:** Sindre Lee, Frode Norheim, Hanne L. Gulseth, Torgrim M. Langleite, Andreas Aker, Thomas E. Gundersen, Torgeir Holen, Kåre I. Birkeland, Christian A. Drevon

**Affiliations:** 1 Department of Nutrition, Institute of Basic Medical Sciences, Faculty of Medicine, University of Oslo, Oslo, Norway; 2 Department of Endocrinology, Morbid Obesity and Preventive Medicine, Oslo University Hospital, Oslo, Norway; 3 Department of Medicine, Division of Cardiology, University of California at Los Angeles, Los Angeles, CA 90095 USA; 4 Vitas Ltd, Oslo Innovation Park, Oslo, Norway; 5 Institute of Clinical Medicine, Faculty of medicine, University of Oslo, Oslo, Norway

Correction to: *Scientific Reports* 10.1038/s41598-018-24976-x, published online 25 April 2018

The Acknowledgements section in this Article is incomplete.

“The MyoGlu trial is registered at ClinicalTrail.gov; NCT01803568. This work was supported by grants from the institute of Basic Medical Sciences, UiO, Johan Throne-Holst Foundation for Nutrition Research, Freia Medical Research Foundation, the “Functional Genomics” and “Infrastructure” programs of the Research Council of Norway and the Southeastern Regional Health Authorities and EU-financed FP7 project (NutriTech grant agreement no: 289511). We thank Anne Randi Enget, Ansgar Heck and Birgitte Nellemann for taking the biopsies, and Tor I Gloppen, Torstein Dalen, Håvard Moen, Marius A Dahl, Guro Grøthe, Egil Johansen, Katrine A Krog, Øyvind Skattebo, Daniel S Tangen, Kristoffer K Jensen, Hans K Stadheim, and Eirin Rise for conducting the human strength and endurance intervention (MyoGlu) under supervision of Professor Jørgen Jensen. Sequencing was performed by PhD Gregor Gilfilan at the Norwegian Sequencing Centre (www.sequencing.uio.no) supported by the Research Council of Norway and the South-eastern Regional Health Authorities.”

should read:

“The MyoGlu trial is registered at ClinicalTrail.gov; NCT01803568. This work was supported by grants from the institute of Basic Medical Sciences, UiO, Johan Throne-Holst Foundation for Nutrition Research, Freia Medical Research Foundation, the “Functional Genomics” and “Infrastructure” programs of the Research Council of Norway and the Southeastern Regional Health Authorities and EU-financed FP7 project (NutriTech grant agreement no: 289511). We thank Anne Randi Enget, Ansgar Heck and Birgitte Nellemann for taking the biopsies, and Tor I Gloppen, Torstein Dalen, Håvard Moen, Marius A Dahl, Guro Grøthe, Egil Johansen, Katrine A Krog, Øyvind Skattebo, Daniel S Tangen, Kristoffer K Jensen, Hans K Stadheim, and Eirin Rise for conducting the human strength and endurance intervention (MyoGlu) under supervision of Professor Jørgen Jensen. Sequencing was performed by PhD Gregor Gilfilan at the Norwegian Sequencing Centre (www.sequencing.uio.no) supported by the Research Council of Norway and the South-eastern Regional Health Authorities. EU Joint Programming Initiative ‘A Healthy Diet for a Healthy Life’ on Biomarkers BioNH FOODBALL (grant number 52905100).”

